# Uses of Personal Health Records for Communication Among Colorectal Cancer Survivors, Caregivers, and Providers: Interview and Observational Study in a Human-Computer Interaction Laboratory

**DOI:** 10.2196/16447

**Published:** 2022-01-25

**Authors:** David A Haggstrom, Thomas Carr

**Affiliations:** 1 VA HSR&D Center for Health Information and Communication Roudebush Veterans Affairs Medical Center Indianapolis, IN United States; 2 Division of General Internal Medicine & Geriatrics Indiana University School of Medicine Indianapolis, IN United States; 3 Center for Health Services Research Regenstrief Institute Indianapolis, IN United States

**Keywords:** personal health record, communication, cancer survivorship, colorectal cancer

## Abstract

**Background:**

Personal health records (PHRs) may be useful for patient self-management and participation in communication with their caregivers and health care providers. As each potential participant’s role is different, their perception of the best uses of a PHR may vary.

**Objective:**

The perspectives of patients, caregivers, and providers were all evaluated concurrently in relation to a PHR developed for colorectal cancer (CRC) survivors.

**Methods:**

We explored group perceptions of a CRC PHR prototype. Scenario-based testing across eight use cases, with semistructured follow-up interviews, was videotaped in a human-computer interaction laboratory with patients, caregivers, and health care providers. Providers included oncologists, gastroenterologists, and primary care physicians. Discrete observations underwent grounded theory visual affinity analysis to identify emergent themes.

**Results:**

Observations fell into three major themes: the network (who should be granted access to the PHR by the patient), functions (helpful activities the PHR enabled), and implementation (how to adopt the PHR into workflow). Patients wanted physician access to their PHR, as well as family member access, especially when they lived at a distance. All groups noted the added value of linking the PHR to an electronic health record, self-tracking, self-management, and secure messaging. Patients and caregivers also saw information in the PHR as a useful memory tool given their visits to multiple doctors. Providers had reservations about patients viewing raw data, which they were not prepared to interpret or might be inaccurate; patients and caregivers did not express any reservations about having access to more information. Patients saw PHR communication functions as a potential tool for relationship building. Patients and caregivers valued the journal as a tool for reflection and delivery of emotional support. Providers felt the PHR would facilitate patient-physician communication but worried that sharing journal access would make the doctor-patient relationship less professional and had reservations about the time burden of reviewing. Strategies suggested for efficient adoption into workflow included team delegation. Establishment of parameters for patient uses and provider responses was perceived as good standard practice.

**Conclusions:**

PHR perceptions differed by role, with providers seeing the PHR as informational, while patients and caregivers viewed the tool as more relational. Personal health records should be linked to electronic health records for ease of use. Tailoring access, content, and implementation of the PHR is essential. Technology changes have the potential to change the nature of the patient-physician relationship. Patients and providers should establish shared expectations about the optimal use of the PHR and explore how emerging patient-centered technologies can be successfully implemented in modern medical practice to improve the relational quality of care.

## Introduction

Personal Health Records (PHRs) have grown in popularity and functionality over time. PHRs are grounded in the idea that patients can improve continuity of care by transporting copies of their records from doctor to doctor. Originally, the term “PHR” stood for “patient-held records” and were paper-based systems [1]. Initially designed by patients, institutions later developed standardized PHR formats to include medication lists, test reports, and physician notes [1-3]. The patient empowerment movement and the internet transformed PHRs into “personal health records” [1,4,5]. Advances in information technology have provided new tools for web-based self-management, communication, and information-sharing to enable patients to play a more active role in their care.

In the United States, patients with chronic disease represented the first target populations for PHRs [4,5]; while their adoption rate is only slightly higher than that in the general population, patients with chronic diseases make greater use of PHR capabilities [6]. Many PHR self-management tools are disease-specific, suggesting that the ideal composition might vary by disease [7-9]; this insight has led to the development of specialized PHRs designed for different chronic diseases, such as diabetes, heart disease, or neurologic disorders [10]. With increases in cancer survival, oncology care has increasingly assumed the characteristics of chronic disease management [11]. To meet the needs of long-term cancer survivorship, the Institute of Medicine (IOM) recommended the development of survivorship care plans to provide a treatment summary and plan for follow-up care, including the potential side effects and long-term consequences of treatment, the timing and content of recommended follow-up, and psychosocial services available in the community [12]. PHRs provide a natural platform on which to address these goals; thus, they have been developed for several cancers [5]. Colorectal cancer (CRC) is the second-most common cause of cancer death in the United States, approximately 147,950 individuals will be diagnosed with CRC and 53,200 will die from the disease in 2020 [13]. All of the general issues addressed by survivorship care plans are of specific relevance to CRC survivors, including follow-up care or surveillance (colonoscopy, carcinoembryonic testing, and abdominal imaging [14]) and potential side effects (eg, radiation proctitis [15] or oxaliplatin neuropathy [16]).

The patient is arguably the key stakeholder, or user, in PHR design. Nonetheless, the proliferation of both synchronous and asynchronous methods of communication has expanded the scope of stakeholders to include both caregivers and health care providers. To create the most effective PHR, the design process needs to account for the needs of all potential stakeholders. Prior studies have examined the perspectives of each stakeholder group individually or focused upon combinations of providers [17] or patients and caregivers [18], or even provider perspectives of caregiver use [19]. Nonetheless, the input of stakeholders such as patients, caregivers, and providers are not commonly considered. Prior qualitative research has shown that patients perceive web-based chronic disease management portals as increasing their access to information and engagement in health care, but improvements in portal design may improve usability and reduce attrition. Caregivers have expressed high interest in portal use to support their roles in interpreting health information, advocating for quality care, and managing medical care [18]. Providers have previously described secure messaging as having particular value for both themselves and their patients; however, providers also expressed concern about the inability of patients to share other types of information with their health care team [17] and the impact on workflow. In this study, the perspectives of patients, caregivers, and health care providers were all evaluated concurrently in relation to a PHR developed for CRC survivors. Patient and caregiver engagement is important for the adoption of PHRs, whereas provider buy-in is critical to the implementation of these technologies in health care settings.

Our key study question was what are the areas of agreement and disagreement among patients, caregivers, and providers with respect to the benefits and appropriate uses of a PHR. Across stakeholders, we explored several questions, including “Who should be provided access to, share information, and communicate with the PHR?” and “What type of patient-generated information should be incorporated into the PHR?” Finally, we asked how the PHR impacts workflow and what best practices may guide the future design and implementation of PHRs for patients with cancer.

## Methods

### Participants

Four to six participants were recruited from each role group (patient, provider, and caregiver) on the basis of a previous study by Nielsen et al [20], suggesting that this number is sufficient to detect the majority of usability problems. Six CRC survivors were recruited from the Roudebush Veterans Affairs Medical Center (RVAMC) oncology clinic in Indianapolis. Provider schedules were reviewed prior to their clinic visit, and then research assistants approached patients in-person at their planned clinic visit. Either during the clinic encounter or later when scheduling the testing session over the telephone, patients were invited to identify a caregiver who could also participate in the session. For inclusion, cancer survivors were required to have a diagnosis of colorectal cancer more than 12 months prior to enrollment. This yearlong interval was chosen to identify patients who were likely to have undergone both surgical and adjuvant therapy so that they could provide feedback on both treatment modalities, as well as to minimize respondent burden upon any patient undergoing active treatment. One caregiver, identified as a family member or friend supporting the cancer survivor’s health needs, was recruited along with each cancer survivor. Seven health care providers were purposefully recruited via email from the RVAMC, including an oncologist, oncology nurse, gastroenterologist, and 4 primary care physicians. In terms of incentives, gift cards were offered in the amount of US $5 to providers and US $25 to patients and caregivers. All participants provided informed consent, and the study protocol was approved by the institutional review board of Indiana University.

### Prototype Design

The initial, web-based CRC PHR prototype was created by a design team of clinical investigators and software developers. The functions of this prototype were informed by the IOM report on Cancer Survivorship [12] and are enumerated in Table 1.

**Figure 1:**
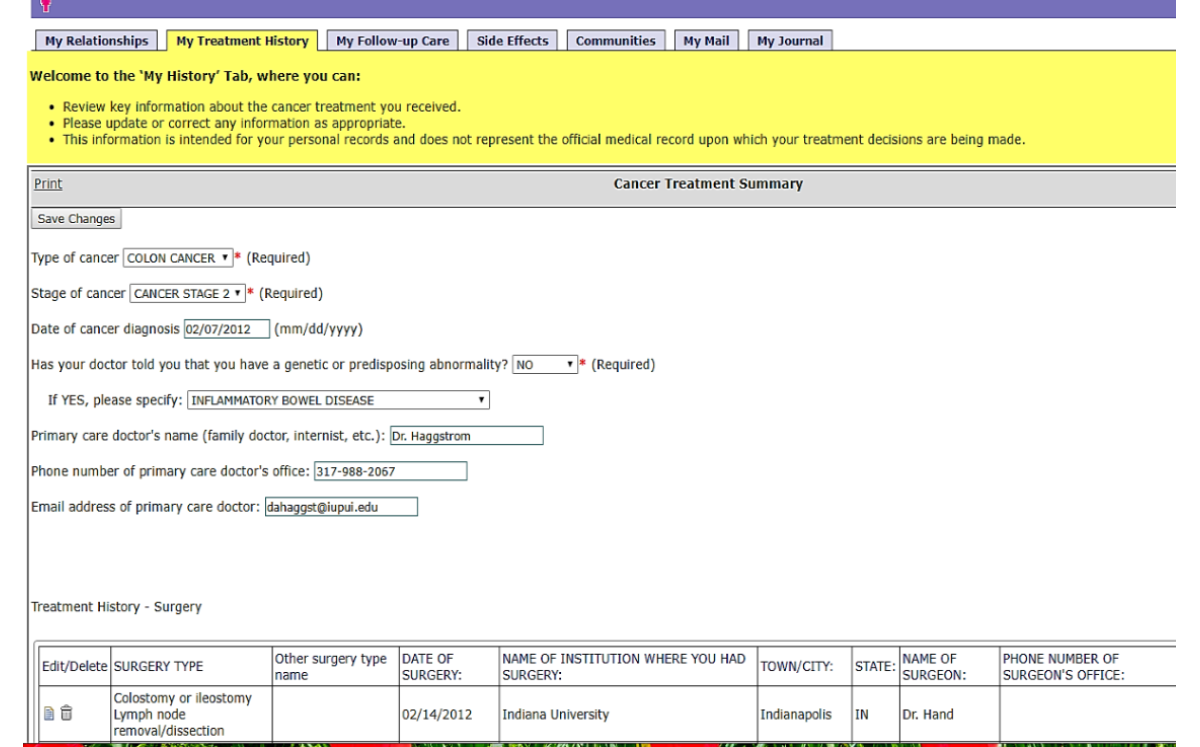
Prototype screenshot created with open-source software—the OpenMRS medical record system platform.

The prototype used a tabbed browser format created with open-source software, the OpenMRS medical record system platform [21] shown in a screenshot in Figure 1.

**Table 1:** Functions of the personal health record of colorectal cancer survivors.

Tab	Functions
My History	Allows review of cancer diagnosis and treatment, including specific type of surgery and adjuvant therapy (chemotherapy, and radiotherapy)
My Follow-up Care	Two tables: a table with recommended surveillance tests based on initial diagnosis and a table of actual tests performed (date, test, and result)
Side Effects	Tailored compendium of possible side effects of treatment and initial, straightforward self-management steps
Communities	Web-based links to cancer information resources and cancer survivor support groups
Relationships	Patients can share access to their personal health records with a set of role-based individuals (provider, caregiver, etc): relationship function enables tiered access to personal (My Mail and My Journal), medical (My History, My Follow-up Care, and Side Effects) or all components of their personal health record
My Mail	Client-based email application enabling secure message exchange
My Journal	Searchable, dated electronic journal, with an ability for in-line comments or responses by individuals to whom a Relationship has been granted

### Interviews and Observations

Using the final PHR version, the participants all completed an individual session in the RVAMC human-computer interaction laboratory. Each session lasted 1 hour and was conducted by a member of the study team with a background in human factors engineering. None of the participants were familiar with the PHR prior to enrollment, nor was any type of tutorial provided before use so as to avoid bias on the basis of user experience, as well as assess the usability or intuitiveness of the interface. An interview guide was developed with expert clinical input from primary care (2) and subspecialty (1) providers, as well as scientific input from individuals with training backgrounds in human factors engineering (1 PhD and 2 Masters). At the outset of each session, all participants underwent the same semistructured interview, including questions concerning experience and expertise with technology. With content tailored to their role (cancer survivor, caregiver, or provider), participants were then given use-case scenarios to perform using the PHR (Table 2) with a PC. The think-aloud design was used during scenario-testing and open-ended follow-up questions were used after each scenario. Concluding questions were then asked, with encouragement to envision a “blue-sky” future or the ideal PHR. The session was videotaped using Morae software so that verbal and visual cues and interactions could be analyzed.

**Table 2:** Use-case testing scenario, with an example of a colorectal cancer survivor.

Task	Description
1A	You recall that your doctor (Dr. Carter) wishes to know if you can grant him access to the electronic tool so he can view all of the information recorded there himself. He tells you to use the email: dcarter@fakeemail.com
1B	You decide you want to grant your spouse access to this electronic tool so she can view all of the information within it herself. Your spouse’s email: myspouse@fakeemail.com
2A	Dr. Carter performed a colonoscopy on January 8, 2020, and found an abnormality, so your doctor has asked you to return in six months. Please record this information using the tool.
2B	After completing the last task, check to see when your next colonoscopy test will be and write your answer in the blank provided: Date of next colonoscopy test:_____________
3A	After your doctor reviews your list of past treatments through this electronic tool, your doctor informs you of a mistake in the records in the electronic cancer toolkit. You were recorded as having been treated with Xeloda, but in reality you were treated with Erbitux. Use the system to update this piece of information.
3B	You want to share the radiation therapy you’ve received with your primary care doctor. Because your doctor cannot gain access to the toolkit, please write all the radiation treatment you’ve received in the spaces provided.
4	After updating your past treatment, please use the system to notify (via email) Greg Armstrong (Lance’s Caregiver), whose email address is garmstrong@fakeemail.com
5	Use the system to record a personal experience involving what it’s like to live with cancer. Please use this opportunity to express yourself, and please entitle it: “My Cancer Experience.”

### Analysis

For each role-based participant group, observations were analyzed using a grounded theory approach to determine the requirements for a CRC PHR and functions prioritized by each group. Two investigators, one of whom was not involved in the original interviews, performed the coding and analysis. The videotapes were broken into single, verbal or non-verbal, observations. The observations were recorded chronologically at the point in the session when they were collected and then grouped by participant role. Data analysis was inductive using the method of constant comparison, an iterative process consisting of an open and focused coding phase [22]. Within each participant role, the observations were coded using visual affinity diagramming [23] with an open-coding scheme. After independent coding by the investigators, coding discrepancies were resolved at consensus meetings. Investigators each read all transcripts and analyzed them for prevalent and recurrent themes. This phase elucidated three overarching themes: the web-based network, its functions, and its implementation. During the focused coding phase, investigators developed additional themes by conducting comparisons within and between transcripts, between themes, and finally across the role-based participant groups. Throughout the analysis, qualitative methods and procedures were used to ensure rigor and validity. These procedures included reflexivity (continually questioning interpretations and returning to the data to verify interpretations), search for alternative interpretations of the data, and depth of description (seeking out the specific details of participants’ words) [24,25].

## Results

### Results Overview

All 6 patients were male, with an average age of 62.2 years (range 54-72 years); 2 of 6 (33%) were African American. Four caregiver spouses were recruited; all were women and half were African American. Of the health care providers, 4 of 7 (57%) were female, 5 (71%) were White, and 2 (29%) were Asian American. Overall, most patients would recommend the use of the PHR to other patients. Patient, caregiver, and provider observations could be grouped into three major themes: the network, its functions, and implementation. The “network” encompassed those who should be granted access to the PHR by the patient, “functions” comprised the helpful activities which the PHR enabled, and “implementation” included how best to implement the PHR into workflow and communication. These themes interrelate dynamically; for example, the types of individuals included in the network (provider, caregiver, or patient) may influence the range of possible uses.

During review, six additional themes emerged within these overarching themes: Network (Access Privileges and Communication), Functions (Self-tracking and Self-management; Journal (Reflection and Communication), and Implementation (Workflow and Future Enhancements).

### Network

#### Access Privileges

The PHR allowed only the patient the ability to grant access to others; many permutations in who should be granted access were observed. All patients wanted physician access to their PHR. Most patients wanted family members to have access to the PHR; when family member access was granted, their spouse was always included. Access for family members was guided by relational closeness, and how important individual family members were to the patient’s care. Without being prompted, 2 different patients suggested that researchers should also be provided access so as to create knowledge in the process of sharing information, expressing a sense of altruism: “probably use if I thought it would help other people.”

#### Communication

*Provider-to-provider communication*: Participants from each stakeholder group saw the value of communication between providers. Patients and providers recommended that health information in the PHR be shared across health care systems. Patients also recommended sharing across different types of providers (both primary care and oncologist). One patient stated that primary care provider access should be “required.”

Another patient suggested a doctor-only network, established without individual patient permissions. Patients suggested a model wherein medical personnel would have one-time access to PHR data in an emergency: “In the past, I had an uncle who died of diabetes and no one knew it but his wife, and she was out of town; he went to the emergency room and they gave him the wrong medicine.”

*Patient-to-provider communication*: The PHR provided at least two opportunities for asynchronous communication between patients and providers through (1) secure email and (2) web-based journal access. Both patients and providers saw potential in the PHR for sharing medical information; patients more often saw potential in the tool for relationship-building. Each stakeholder group described how the optimal mode of communication varied by its purpose. Email was described as acceptable for simple messages, but not for complex or sensitive topics (eg, bad news) or issues requiring an immediate response, which were considered to be most appropriately communicated in person or by telephone.

*Patient-to-caregiver communication*: Among patients and caregivers, PHRs were perceived as adding value when family members lived at a distance from the patient. This distance could range from out-of-the-home to out-of-state. Patients discussed how providing caregivers access to the PHR could inform caregivers about possible side effects or the symptoms patients were experiencing. Although most spouses agreed with the desirability of access to the patient’s PHR, one described their access to the patient’s PHR as “an invasion of privacy.”

### Functions

#### Self-tracking and Self-management

Patients and caregivers saw the information recorded in the PHR as a useful memory tool because they “can’t remember everything.” One caregiver noted that this would be “great for keeping up with what is happening with 10 doctors.” All stakeholder groups observed that the PHR would enable the patient and caregivers to record and track future testing, leading them to be “more engaged.”

Patients and providers saw potential in the PHR for self-management. One patient said, “It would be nice to be able to keep up with how your treatment’s going, kind of knowing if you’re…getting better…A lot of times when you have cancer you always have a question mark over your head – where am I?” One provider discussed how the PHR could stimulate patients to ask questions. A caregiver noted that information from the PHR could be printed and brought to doctor appointments to address key issues. Patients and providers saw the potential for the PHR to track symptoms and perhaps deliver self-care approaches. Given the network of relationships that the PHR facilitated, patients saw an opportunity for collective self-management of their problems, facilitating help from caregivers.

Providers had reservations about patients viewing “raw data.” They felt that patients were not prepared to interpret the data, which led to confusion and anxiety, and that inaccurate data in the record might upset patients. Patients and caregivers did not raise any downsides to having access to more information. Regarding inaccurate information in the PHR, patients expressed greater interest in how errors might be corrected rather than who would be to blame. None of the patients raised concerns about privacy or security of the PHR, but caregivers and providers expressed such concerns.

#### Journal: Reflection and Communication

Patients viewed the journal as a tool for reflection where they could record their personal thoughts, emotions, symptoms, and “vent” about frustrations. If shared, information recorded in the journal was seen as potentially reducing a sense of isolation: “Cancer is kind of a lonely illness to have; they can talk back and forth and share their experience of what they are going through and what medicines are working for them.” Patients and caregivers saw the PHR as providing a way for others, including the health care provider, to better “understand the patient’s issues”.

In addition to self-reflection, patients and caregivers viewed the journal as a tool for communication and a way for patients to receive support from others. The “comments” section in the journal appeared to them to make bidirectional communication possible between patients and providers. For more stoic individuals, the journal was seen as offering a tool to communicate in written form what they might have trouble expressing verbally. One caregiver noted that a patient may withhold information about prognosis to “protect” family members, and the journal may enable greater sharing of information: “helps with hope if we know what to expect.” Another caregiver suggested more multimedia resources in the PHR; for example, “songs or movies” that could help start conversations.

Two providers shared the view that the PHR would facilitate patient-physician communication, allow “sympathy,” and help the physician understand the patient “holistically.” However, another provider worried that sharing journal access would make the doctor-patient relationship “less professional.” Most providers were concerned about the time burden of processing a large amount of unstructured information. Potential malpractice liability owing to the provider having journal access was also raised, although a provider commented “you can’t spend life worrying about lawsuits.”

### Implementation

#### Workflow

All stakeholder groups would prefer the PHR to be tethered to the patient’s electronic health record (EHR), and did not see themselves as performing manual data entry. One participant indicated concern that only young or “techie” patients would be able to reliably use the PHR. For certain types of data, providers did not trust the accuracy or completeness of patient-entered information, although one provider stated that “patients should record their own values so they will be more involved.”

The time burden of accessing the PHR was a common concern among providers. Strategies suggested for efficient adoption into workflow included nurse delegation; for example, email could be used for nurse-directed symptom management. Each stakeholder group believed the PHR should be well-integrated with other technologies to avoid creating multiple locations to access electronic health records or check email. PHR training was also perceived as necessary. Establishing parameters for patient uses and provider responses was considered good standard practice.

For email, providers were again concerned about the time burden but recognized that email could be both a “responsive” and “efficient” tool (eg, sending test results) for asynchronous communication. Patients expressed sensitivity to the time burdens of providers without prompting from the interviewer, suggesting that email may reduce the number of telephone calls, and expressing the opinion that email was more likely to reach their doctors.

Most providers considered email more efficient than the journal. A few providers indicated they would read the journal, but only if directed by patient request to a specific entry. For the journal, one provider suspected patients would record too much extraneous detail, thus making real issues harder to find. Providers suggested several types of structured information patients could enter, including review of systems, symptoms, and pain scores. Organizing tools, such as the use of subject headings and natural language processing, were suggested.

#### Future Enhancements

Patients and caregivers were interested in several specific enhancements to the PHR. They sought more guidance in accessing support groups and information regarding complementary medicine. Patients were interested not only in disease information, but also healthy lifestyle resources, especially nutrition. More capabilities concerning medication management were suggested, particularly a list of medications and side effects attributable to each chemotherapy agent. Other desired functions were the ability to refill medications, make appointments, and carry the PHR on portable devices.

## Discussion

### Principal Findings

The integration of multiple stakeholder perspectives regarding the potential use of a PHR for cancer survivors was a key strength of our study. Patients, caregivers, and providers all have unique roles and offer particular insights into the PHR’s potential to meet the needs of cancer survivors. All stakeholder groups perceived the PHR to be a valuable tool and would recommend this patient-centered technology to others diagnosed with cancer. Several areas of agreement emerged across different stakeholder groups. First, the broader the network of users provided access, the better. As in other studies, a majority of patients wanted clinician access to the PHR [26], especially primary care physicians and oncologists. Stakeholder groups also recommended that networks bridge multiple health care systems. Essentially, participants were articulating a model of health information exchange, which shared electronic treatment information across multiple organizations, although perhaps they were unfamiliar with existing technical architectures or platforms to accomplish information-sharing [27].

Patient preference for access among individuals who were not clinicians was less universal and connected with the closeness of personal relationships and geographic proximity [8,28]. In patients with cancer, it may be especially beneficial for caregivers to be given access to the PHR [29], although such access needs to be balanced against the countervailing principles of patient privacy so as to prevent unwanted disclosures; for example, stigmatized conditions or billing information [30]. Overall, patients valued the ability to control access to the record on an individual basis [8]. While other systems allow the patient to control who else can access the PHR [31], this CRC PHR also enables the patient to control what domains of the records (medical versus personal) are accessed by whom. Consequently, the patient can share information with each individual provider and caregiver at the level that they choose. A patient could selectively provide access to the journal to family members owing to the personal nature of the content. Alternatively, a patient may choose to provide only medical providers with access to the treatment summary owing to its clinical nature and to preserve their privacy. But instead of making a priori assumptions about what decisions patient will make, the PHR provides patients with autonomy to tailor these decisions on the basis of their preferences for disclosure.

Another area of wide agreement among all stakeholder groups was the use of the PHR for information-sharing. Pragmatically, health information delivered through the PHR may increase patient recall and prompt questions at follow-up visits. By enabling patients to review their health information beforehand, and potential test recommendations, patients may be more prepared and activated [32,33] at physician visits. Furthermore, leveraging its information-sharing and communication functions, the PHR may serve as a foundation for collaborative decision-making and shared decisions.

Areas of disagreement were also noted among stakeholder groups, particularly between patients or caregivers and health care providers. Patient and caregivers both saw the value of the PHR in relationship-building. Information the patient shared about their personal cancer experience, especially through the journal function, was viewed as a way to be better understood as a whole person. Previous studies of narrative medicine suggest that patients’ written stories of how illness has affected them can help them rediscover personal identity [34] and even improve patient outcomes [35]. Providers were concerned about the shift such web-based technologies could bring about in their professional roles. Previous research has outlined a mixed picture of social networking technologies. Social media use by patients led to more equal communication between the patient and provider, but increased doctor-switching [36]; patient-provider relationships may also be more harmonious owing to the opportunity to release negative emotions on the internet, but others have found suboptimal interactions between the patient and health care professional if providers do not agree with the information provided, or even feel their expertise challenged [37].

The messaging function, especially to communicate with their doctor, was highly valued by patients in this and other PHR studies [7,8,28,38]. Nonetheless, despite studies showing the feasibility of web-based patient-doctor communication [39], providers were concerned about time burden, data security, and privacy. While sensitive to time concerns, data privacy and security issues were not independently raised by the patients, which suggested that they were much more focused on the potential benefits than these mitigating risks. Consensus across stakeholder groups was easier to find on general guidelines for email use; for example, simple messages for nonemergent issues.

A related area of disagreement between patients and providers was the perceived value of sharing unstructured information. Patients saw the ability to construct meaning and relate personal experiences from sharing information in more qualitative forms, but only a minority of providers shared this view. Providers saw the same information as potentially inaccurate, a time burden, and source of medical liability. Structured information of all types was suggested by providers, including symptom and pain scores. Such discrete information may be more manageable for tracking and quality improvement purposes, as well as secondary research; however, the use of standardized instruments reduces the expressive content of the information and the patient’s ability to articulate their unique circumstances.

Our findings deliver several key messages to be considered in the future design and implementation of patient-centered technologies.

#### PHRs Should Be Linked to EHRs

In free-standing PHRs, data entry would need to be performed by either the patient or the health care provider. Neither cancer survivors nor health care providers could see themselves as having adequate time to enter such data into the PHR. Moreover, health care providers did not have full confidence in the accuracy of patient-entered data. Based on the medical literature, their skepticism is warranted; for example, patients tend to overreport the receipt of cancer screening and underreport screening intervals [40]. Tethering or linking PHRs to EHRs would also enable the wide range of networking envisioned by patients. Providers who are already using EHRs could more readily be provided with access to PHRs. Through patient health information exchanges, multiple health care systems can be digitally connected. Broadband internet access serves as a barrier to PHR use among underserved populations and policy changes such as patient subsidies [41], and increased rural broadband infrastructure [42] also have a role to play in improving the health information ecosystem.

#### Emergency Care Is a Convincing Use Case

In this and other studies, patients expressed interest in allowing emergency care providers to temporarily access the PHR [43]. Cancer survivors and other chronic disease PHR users valued this emergency access, over privacy concerns [8,43]. A patient in this study and another study [8] related occasions where an unfavorable outcome resulted from the inability of emergency providers to access records. This patient-requested feature could be incorporated into future PHRs by a single-use key code, carried on the patient or held by an emergency contact.

#### Tailoring Is Essential

Tailoring across multiple dimensions of the PHR is possible, including access, content, and implementation approaches. As a matter of patient-centered principle, the patient was placed in control of PHR access; cancer survivors can then choose who to invite as well as what types of information those individuals can access. This flexible design appeared to be well-received, and patients reflected thoughtfully about to whom and why they would provide access. In our study and others, patients also wanted tailored guidance in searching for high-quality disease information and local support groups [44]. Of course, barriers to PHR adoption remain, and our group has previously identified barriers to use among a population of patients with CRC of similar age (mean age 58 years), including difficulty with system log-in, lack of computer literacy, and difficulty self-entering patient information [45].

The organizational contexts in which patients are seen, and in which PHRs will be administered, are quite heterogeneous. The US health care system has a multiplicity of practice environments, including academic and community, private and public, hospital- and office-based, and single and multispecialty groups. The structure and workflow of individual practices will play a large role in the optimal approach to implementation. Helpful guidance was suggested by our participants, including the use of support staff and best practices in the use of emails. Observations collected in multiple clinical settings would more fully inform other approaches worth consideration for dissemination.

Structured information was entered to summarize the patient’s treatment and surveillance testing, and unstructured information was communicated in the journal and messaging system. Preferences for different types of information diverged between patients and providers. To resolve these varying perspectives, negotiation may be necessary between patients and providers to strike what both view as a fair balance. Different PHR systems may be tailored over time to reflect these compromises in the types of information delivered and received.

#### Technology Changes Have the Potential to Change the Nature of the Patient-Physician Relationship

Patients almost universally valued the participation of health care providers in the PHR system. However, providers expressed reluctance about an open-ended engagement, especially in the case of patients expressing personal feelings or experiences in unstructured formats. While technology may initially appear to be a tool to facilitate patient-physician communication, it is worth considering how new tools such as the PHR could potentially change not only the mode, but also the content and qualities of communication. Broader sharing of the cancer experience may provide the opportunity for providers to more deeply understand their patients’ identities. Further, the low adoption rate of PHRs [46] might be improved if these technologies enabled such high-quality, meaningful communication between patients and providers; a prior systematic review confirms that patients highly value using portals for communication with providers [47]. However, this more personal connection may narrow the professional distance between patients and providers in such a way that not all parties are comfortable. As more professional and social experiences transition to web-based digital platforms, the patient-physician relationship at the center of medicine may evolve in other unexpected or unintended ways. To our knowledge, our study results uniquely highlight the trade-offs and tensions that web-based technologies may introduce into the domain of patient-provider communication. Previous studies have perhaps focused upon the impact of shared records upon workflow [48], but not necessarily the nature of the patient-physician relationship itself. Our research design of incorporating both patient and provider perspectives was key to the discovery of these findings.

### Limitations

This study was limited by its size, although owing to recurrent themes in the analysis, investigators believed that thematic saturation had been reached. Further, many of the main findings were consistent across different subject groups. The participant population was completely male; while males represent the strong majority of the US veteran population (>90%), female veterans should be aggressively recruited in future studies. The age distribution was also representative of patients diagnosed with colorectal cancer. Future studies should consider other patient groups and cancer types. Finally, no major EHR companies have deployed oncology patient portals that make possible the clinically tailored care delivered by the PHR tested here. Hence, we focused on general PHR issues (networking and implementation) and functions (self-management and journaling) relevant across portals. However, we believe that tailored, disease-focused PHRs have the potential to deliver greater clinical value to patients and providers; therefore, they represent a future model for technology design when the industry’s business case can better support the degree of specialization required.

### Conclusions

PHR perceptions are role-dependent, but there is marked consensus on many aspects of PHR design among stakeholders. This suggests that a single, integrated tool can be designed to meet several identified patient needs, including self-tracking and self-management, as well as more informed and shared medical decisions. Providers have unique concerns about the increased time burden and the accuracy of patient-entered data, and more fundamentally, how web-based communication tools may change the nature of the physician’s professional role. Patients perceive these tools as a potential pathway to personal understanding that can deepen their relationships with doctors. Nonetheless, to realize this promise, patients and caregivers may need to search for and encourage health care providers to partner with them in exploring how emerging patient-centered technologies can be successfully implemented in modern medical practice to improve the relational quality of care.

## Abbreviations

CRC: colorectal cancer
EHR: electronic health record
IOM: Institute of Medicine
PHR: personal health record
RVAMC: Roudebush Veterans Affairs Medical Center
